# Impact of early telemedicine follow-up on 30-Day hospital readmissions

**DOI:** 10.1371/journal.pone.0282081

**Published:** 2023-05-22

**Authors:** Anne Grauer, Talea Cornelius, Marwah Abdalla, Nathalie Moise, Ian M. Kronish, Siqin Ye

**Affiliations:** 1 Department of Medicine, Columbia University Irving Medical Center, New York, New York, United States of America; 2 Center for Behavioral Cardiovascular Health, Columbia University Irving Medical Center, New York, New York, United States of America; 3 Division of Cardiology, Columbia University Irving Medical Center, New York, New York, United States of America; Uniwersytet Jagiellonski w Krakowie Biblioteka Jagiellonska, POLAND

## Abstract

**Introduction:**

Telemedicine is increasing in popularity but the impact of this shift on patient outcomes has not been well described. Prior data has shown that early post-discharge office visits can reduce readmissions. However, it is unknown if routine use of telemedicine visits for this purpose is similarly beneficial.

**Materials and methods:**

We conducted a retrospective observational study using electronic health records data to assess if the rate of 30-day hospital readmissions differed between modality of visit for primary care or cardiology post-discharge follow-up visits.

**Results:**

Compared to discharges with completed in-person follow-up visits, the adjusted odds of readmission for those with telemedicine follow-up visits was not significantly different (odds ratio [OR] 0.96, 95% confidence interval [CI] 0.61 to 1.51, P = 0.86).

**Conclusions:**

Our study showed that 30-day readmission rate did not differ significantly according to the modality of visit. These results provide reassurance that telemedicine visits are a safe and viable alternative for primary care or cardiology post-hospitalization follow-up.

## Introduction

During the COVID pandemic the landscape of ambulatory care changed. Routine appointments transitioned from traditional in-person visits to a combination of telehealth and in-person in order to decrease the spread of disease [1, 2]. However, the effect of this transition on clinical outcomes is still emerging.

Previously, early follow-up visits with primary care and cardiology clinics have been shown to decrease readmission after hospitalizations [3–5]. However, it is unknown if the routine use of telemedicine visits for this purpose is similarly beneficial. While telemedicine will continue to be an important aspect of the healthcare landscape after the Covid-19 pandemic, evidence is still emerging for whether telemedicine post-discharge visits lead to similar, better, or worse outcomes with regards to readmissions, compared to in-person visits. In theory, telehealth can improve access to care, improve medication reconciliation and ease transition from hospital to home environment which could lead to a decrease in hospital readmissions. In fact, previous studies have shown post-discharge telehealth appointments are more likely to be completed than in-person visits [6, 7]. One prior study which randomized patients to an integrated system of remote patient monitoring vs traditional in-person visits also showed improved medication reconciliation, adherence and patient satisfaction in the telehealth arm [8]. However, there has also been concern for diagnostic accuracy during telemedicine visits [9], which are less conducive for accurate physical examination and have been shown to have lower rates of testing [10].

A 2022 study examined heart failure discharges from a large health system, and found those who received early (within 14 days) follow-up had similar rates of readmission regardless of whether the follow-up was in-person or telemedicine [11]. We sought to further elucidate the impact of telemedicine follow-up visit on readmissions for hospital discharges in general, focusing on primary care and cardiology post-discharge settings where the association between early-follow-up and reduced readmission has been best described [12, 13]. Specifically, using data from the electronic health records (EHR) of a large urban academic medical center, we conducted a retrospective analysis of patients with primary care or cardiology follow-up visits within 7 days of discharge, comparing the rate of 30-day readmissions between those who attended telemedicine follow-up visits, in-person follow-up visits, or those who were scheduled but did not attend.

## Materials and methods

### Data extraction and patient sample

After approval by the Columbia University Institutional Review Board (IRB), we queried our EHR (Epic Systems, Verona, Wisconsin, 2020) to identify all adult, non-obstetric, and non-Covid related discharges from Columbia University Irving Medical Center (CUIMC) and NewYork-Presbyterian Allen Hospital between 6/1/2020 to 11/30/2020. The requirement of informed consent was waived in accordance with IRB guidelines. This time period was selected as both in-person and telemedicine outpatient care became readily available again following the initial Covid-19 peak in New York City.

For each visit, we extracted demographic and clinical information including date of birth, sex, race, ethnicity, primary payer, admission source, follow-up specialty, discharge with home-services, type of visit (in-person, or telemedicine via video or telephone) and International Classification of Disease, 10^th^ Revision (ICD-10) codes associated with the visit.

### Outcomes definition

**Fig 1:**
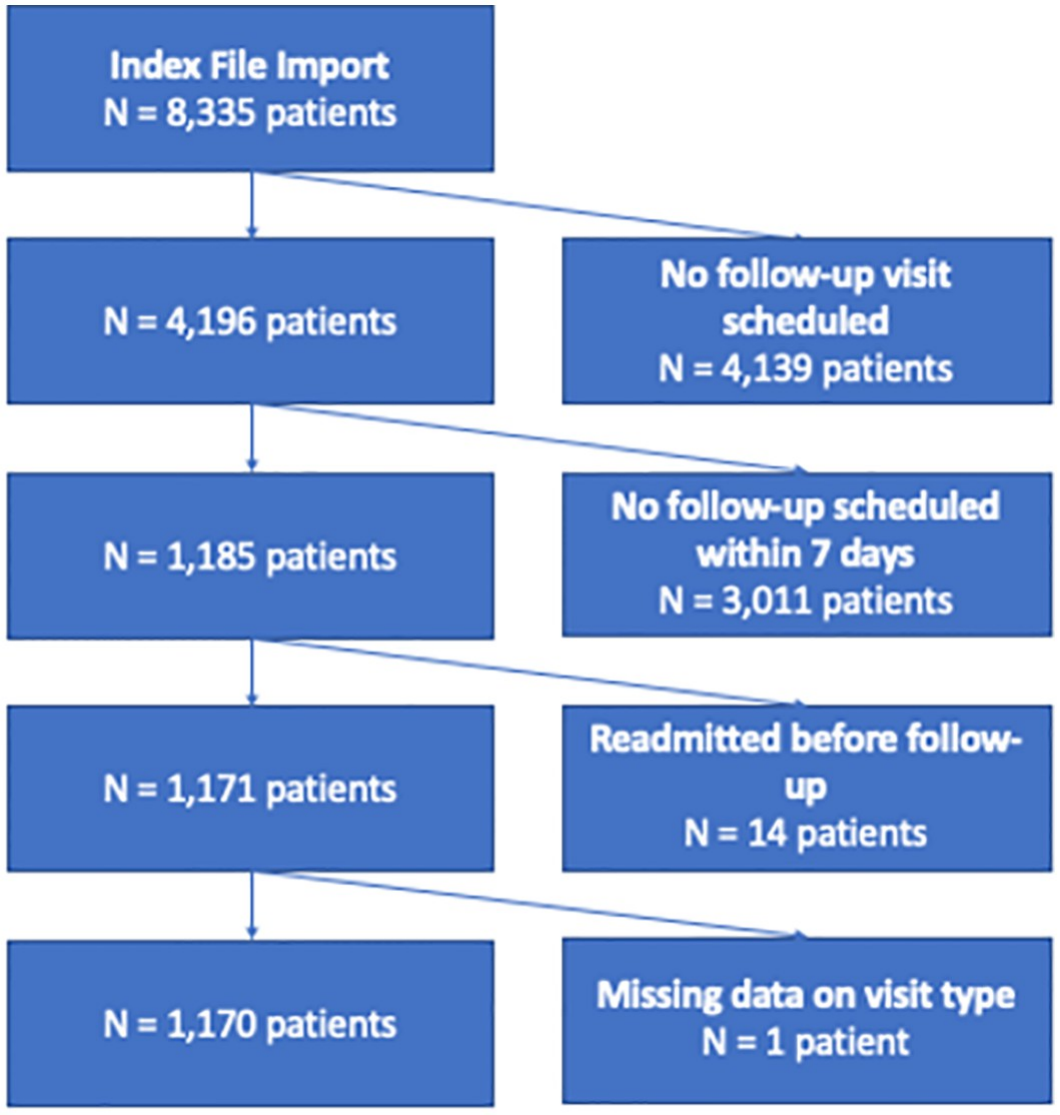
STROBE flowchart demonstrating sample selection process.

We included for analysis all discharges to home setting that also had a completed or cancelled in-person or telemedicine (including video and telephone-only) primary care (defined as an internal medicine or family medicine visit) or cardiology visit within 7 days of discharge; discharges that had readmissions prior to the scheduled follow-up visit or represented a 30-day readmission event were excluded (Fig 1). The primary outcome was readmission within 30 days of the discharge date.

### Telemedicine use

The primary predictors were modality and status of primary care or cardiology visits within 7 days of the discharge date, including 1) completed in-person follow-up visit, 2) completed telemedicine follow-up visit, and 3) scheduled visit with no-show/cancellation. Specifically, we defined a follow -up visit as telemedicine if it was a scheduled visit conducted using video technology or using telephone, based on Epic scheduling data.

### Covariates

We identified clinical and demographic characteristics with known associations with 30 day readmission rate^s^ including a diagnosis of pneumonia, heart failure, or acute myocardial infarction through visit-associated ICD-10 codes [14]. We additionally adjusted for age, sex, race/ethnicity, primary insurance, home services at discharge, admission source being from ED, follow-up visit specialty (cardiology versus primary care), and Elixhauser Comorbidity Index calculated using discharge ICD-10 codes [15].

### Statistical analysis

PROC GLIMMIX models with a random intercept, binomial distribution, and logit link function were implemented in SAS v. 9.4 to test the association of visit status (complete, telehealth; complete, in-person; not complete) with the odds of hospital readmission within 30 days of discharge. Unadjusted P-values are reported, as are odds ratios adjusting for covariates listed above. Unadjusted comparisons of covariables by visit status were also tested using multilevel models to account for nesting within patients.

## Results

### Patient population and baseline characteristics

A total of 1,229 index discharges from 1,170 patients were included for analysis. Of these, 379 (31%), 457 (37%), and 393 (32%) discharges had telemedicine, in-person or cancelled/no-show primary care or cardiology follow-up visits within 7 days of discharge, respectively. Of 393 patients who had a cancelled/no show visit 210 (53.4%) were scheduled as an in-person visit and 183 (46.6%) were scheduled as a telemedicine visit. Furthermore, 227 (57.8%) of 393 patients who had a cancelled/no show visit subsequently completed a visit within 30 days; of these, 81 (35.7%) were telemedicine, and 146 (64.3%) were in-person. As seen in Table 1, groups differed by sex, insurance, race/ethnicity, follow-up visit specialty, presence of congestive heart failure diagnosis, admission source, and Elixhauser Comorbidity Index.

**Table 1:** Demographic and clinical characteristics of patients by modality of post-discharge follow-up visit within 7 days.

	Telemedicine n = 379	In-Person n = 457	Cancelled/no show n = 393	p-value
**30-Day Hospital Readmission**	55 (14.5%)	67 (14.7%)	66 (16.8%)	0.61
**Age**	63.3 (16.4)	64.2 (15.4)	62.8 (16.9)	1.00
**Sex**				
Male	159 (42.0%)	289 (63.2%)	210 (53.4%)	< .001
Female	220 (58.0%)	168 (36.8%)	183 (46.6%)	
**Race/Ethnicity**				
White, non-Hispanic	48 (12.7%)	118 (25.8%)	59 (15.0%)	< .001
Hispanic, non-White	197 (52.0%)	144 (31.5%)	170 (43.3%)	
Black, non-Hispanic	54 (14.3%)	56 (12.3%)	66 (16.8%)	
Asian, Hawaiian & Pacific Islander	5 (1.3%)	7 (1.5%)	5 (1.3%)	
Other / Declined / Unknown	75 (19.8%)	132 (28.9%)	93 (23.7%)	
**Primary Health Insurance**				
Commercial	43 (11.4%)	94 (20.6%)	75 (19.1%)	< .001
Medicare	198 (52.2%)	263 (57.6%)	205 (52.2%)	
Medicaid	138 (36.4%)	100 (21.9%)	113 (28.8%)	
**Home Services**	134 (35.4%)	141 (30.9%)	121 (30.8%)	0.30
**Follow-Up Visit Specialty**				
Primary Care	309 (81.5%)	124 (27.1%)	218 (55.5%)	< .001
Cardiology	70 (18.5%)	333 (72.9%)	175 (44.5%)	
**Hospitalization Reason** ^a^				
Heart Failure	60 (15.8%)	116 (25.4%)	73 (18.6%)	< .01
Pneumonia^b^	14 (3.7%)	29 (6.4%)	16 (4.1%)	.15
Acute Myocardial Infarction	18 (4.8%)	36 (7.9%)	27 (6.9%)	.19
**Admission Source**				
Other	130 (34.3%)	249 (54.5%)	164 (41.7%)	< .001
Emergency Department	249 (65.7%)	208 (45.5%)	229 (58.3%)	
**Elixhauser Comorbidity Index**	3.7 (1.9)	4.2 (2.2)	3.8 (2.2)	< .001

^a^Determined by presence of ICD-10 codes in principal, severe or moderate positions for hospital diagnoses

^b^For pneumonia, discharges for Covid-19 related pneumonias were excluded based on ICD-10 codes.

### 30 day readmission rates

The 30-day readmission rate for discharges with in-person, telemedicine, or cancelled/no-show follow-up visits within 7 days were 15%, 15%, and 17% (P = 0.61), respectively (Table 1). Compared to discharges with completed in-person follow-up visits, the adjusted odds of readmission for those with telemedicine follow-up visits was not significantly different (odds ratio [OR] 0.96, 95% confidence interval [CI] 0.61 to 1.51, P = 0.86), nor were the odds of readmission for those with cancelled/no-show follow-up visits (OR 1.17, 95% 0.78 to 1.74, P = 0.46) (Table 2).

**Table 2:** Predictors of 30-day hospital readmissions as determined using a multivariable, generalized linear mixed model.

	OR (95% CI)	p-value
**Visit Modality**		
In-person	Ref	Ref
Telemedicine	0.96 (0.61, 1.51)	0.86
No show/Cancellation	1.17 (0.78, 1.74)	0.46
**Age (per 10-year increase)**	0.90 (0.79, 1.03)	0.12
**Sex**		
Male	REF	REF
Female	0.96 (0.69, 1.35)	0.83
**Race/Ethnicity**		
Non-Hispanic White	REF	REF
Hispanic, non-White	0.96 (0.59, 1.57)	0.88
Non-Hispanic Black	0.94 (0.53, 1.69)	0.84
Asian, Hawaiian & Pacific Islander	0.66 (0.14, 3.14)	0.60
Other / Declined / Unknown	0.82 (0.49, 1.39)	0.47
**Primary Insurance**		
Commercial	REF	REF
Medicare	1.44 (0.83, 2.50)	0.20
Medicaid	1.76 (1.01, 3.05)	0.05
**Home Services**	1.21 (0.84, 1.74)	0.30
**Follow-Up Visit Specialty**		
Family or Internal Medicine	REF	REF
Cardiology	0.94 (0.63, 1.41)	0.78
**Hospitalization Reason**		
Heart Failure	1.06 (0.70, 1.60)	0.79
Pneumonia^a^	0.72 (0.33, 1.58)	0.42
Acute Myocardial Infarction	1.56 (0.88, 2.79)	0.13
**Admission Source**		
Other	REF	REF
Emergency Department	1.40 (0.98, 1.99)	0.07
**Elixhauser Comorbidity Index**	1.23 (1.13, 1.34)	< .001

^a^For pneumonia, discharges for Covid-19 related pneumonias were excluded based on ICD-10 codes.

## Discussion

In our study of patients discharged from an academic medical center during the period immediately following the widespread telemedicine adoption, we found that those who used telemedicine follow-up visits early after discharge were no more likely to be admitted within 30 days than those whose early follow-up utilized in-person visits. These results provide reassurance that telemedicine visits, which may offer more convenience to recently hospitalized patients, is a safe and viable alternative to post-hospitalization follow-up.

Our study is among the first to examine the impact of early primary care telemedicine follow-up on 30-day readmission rates. Our results are consistent with a recent study which randomized patients to remote patient monitoring programs and compared 30-day readmission rates with a traditional follow-up approach [8]. This study, completed prior to COVID-19, demonstrated that although patients who were randomized to the intervention group were more likely to have medication adherence and welcomed the intervention, there were no statistically significant differences in readmission rates. However, our study differs by examining the routine use of telemedicine for early discharge visits, and extends the literature regarding the safety of this approach.

Our findings are also consistent with a recent study which showed that during the Covid-19 pandemic, patients with heart failure who received outpatient follow-up within 14 days of discharge had similar readmission rates regardless of whether the follow-up was in-person or via telemedicine [11]. Similarly, another recently published study examined the association of telemedicine use for post-discharge follow-up during the COVID-19 pandemic, and found that telemedicine visits were more likely to be completed (84.0% vs 61.5%, p<0.0001) than in-person visits [6]. Specifically, they found an increase in visit completion rates in Black patients from 52% to 70% with the rise of telemedicine usage. The study concluded that telemedicine can narrow disparities and improve access to care in minority populations. In this current analysis we found those who had Medicaid insurance represented 36% of those who had a telemedicine visit vs 22% of those who had an in-person visit and those who identified as minorities including Hispanic, non-White and Black, non-Hispanic represented a greater proportion of those who utilized telemedicine to access care, supporting the potential for telemedicine to decrease disparities in access to care. Further research should specifically focus on the impact of telemedicine usage on health outcomes in minority populations.

In addition to post-discharge care, the effect of routine follow-up telemedicine visits on patient outcomes has been promising and has pointed towards an increase in quality of care [16–22]. For example, in a large cohort of 526,876 patients, those who utilized a combination of telemedicine and in-person visits vs only in-person visits, were more likely to achieve many quality outcomes; undergo cancer screenings, receive guideline-based lab testing, be prescribed guideline-based preventative medications and achieve blood pressure control [16]. The ideal timing interval of telemedicine vs in-person primary care visits and how the two modalities best complement each other warrants further exploration.

Our study has several limitations. Our retrospective design is necessarily hypothesis generating, and we cannot exclude unmeasured confounders such as how patients were selected for telemedicine follow-up visits. Additionally, due to limitations in study design we were unable to study the long term effects of visit modality on patient outcomes, such as all-cause mortality. However, previous studies have shown that 30-day readmission rates are an independent risk factor for all-cause mortality, regardless of severity index and primary diagnosis [23]. We may also be underpowered to detect small differences in 30-day readmission rates. Indeed, the non-statistically significant increase in readmission rate for those with no-show/cancelled follow-up visits may be due to sample size and inadequate power. Because we used EHR data for our analysis, we may also miss readmission events or deaths that occur outside our system, and we were not able to adjust for competing risks. Future studies, including larger randomized controlled studies, will need to confirm our findings and identify best approaches to utilizing telemedicine for post-hospitalization follow-up care.

Our study, which is among the first to examine the impact of routine use of telemedicine visits for early post-hospital follow-up that included all patients discharged from the medicine services regardless of specific diagnoses, showed that 30-day readmission rate did not differ significantly according to the modality of visit. These results provide reassurance that telemedicine visits, which may offer more convenience to recently hospitalized patients, is a safe and viable alternative for post-hospitalization follow-up in cardiology and primary care clinics.

## References

[1] Weiner JP, Bandeian S, Hatef E, Lans D, Liu A, Lemke KW. In-Person and Telehealth Ambulatory Contacts and Costs in a Large US Insured Cohort Before and During the COVID-19 Pandemic. JAMA Netw Open. 2021;4(3):e212618. doi: 10.1001/jamanetworkopen.2021.2618 33755167 PMC7988360

[2] Mann DM, Chen J, Chunara R, Testa PA, Nov O. COVID-19 transforms health care through telemedicine: Evidence from the field. J Am Med Inform Assoc. 2020;27(7):1132–1135. doi: 10.1093/jamia/ocaa072 32324855 PMC7188161

[3] Wiest D, Yang Q, Wilson C, Dravid N. Outcomes of a Citywide Campaign to Reduce Medicaid Hospital Readmissions With Connection to Primary Care Within 7 Days of Hospital Discharge. JAMA Netw Open. 2019;2(1):e187369. doi: 10.1001/jamanetworkopen.2018.7369 30681708 PMC6484580

[4] Vernon D, Brown JE, Griffiths E, Nevill AM, Pinkney M. Reducing readmission rates through a discharge follow-up service. Future Healthc J. 2019;6(2):114–117. doi: 10.7861/futurehosp.6-2-114 31363517 PMC6616175

[5] Metra M, Gheorghiade M, Bonow RO, Dei Cas L. Postdischarge assessment after a heart failure hospitalization: the next step forward. Circulation. 2010;122(18):1782–1785. doi: 10.1161/CIRCULATIONAHA.110.982207 20956215

[6] Bressman E, Werner RM, Childs C, Albrecht A, Myers JS, Adusumalli S. Association of Telemedicine with Primary Care Appointment Access After Hospital Discharge. J Gen Intern Med. 2022;37(11):2879–2881. doi: 10.1007/s11606-021-07321-3 35018569 PMC8751457

[7] Gorodeski EZ, Moennich LA, Riaz H, Jehi L, Young JB, Tang WHW. Virtual Versus In-Person Visits and Appointment No-Show Rates in Heart Failure Care Transitions. Circ Heart Fail. 2020;13(8):e007119. doi: 10.1161/CIRCHEARTFAILURE.120.007119 32762457

[8] Noel K, Messina C, Hou W, Schoenfeld E, Kelly G. Tele-transitions of care (TTOC): a 12-month, randomized controlled trial evaluating the use of Telehealth to achieve triple aim objectives. BMC Fam Pract. 2020;21(1):27. doi: 10.1186/s12875-020-1094-5 32033535 PMC7007639

[9] Gandhi TK, Singh H. Reducing the Risk of Diagnostic Error in the COVID-19 Era. J Hosp Med. 2020;15(6):363–366. doi: 10.12788/jhm.3461 32490798 PMC7289509

[10] Yuan N, Pevnick JM, Botting PG, Elad Y, Miller SJ, Cheng S, et al. Patient Use and Clinical Practice Patterns of Remote Cardiology Clinic Visits in the Era of COVID-19. JAMA Netw Open. 2021;4(4):e214157. Published 2021 Apr 1. doi: 10.1001/jamanetworkopen.2021.4157 33818619 PMC8022216

[11] Xu H, Granger BB, Drake CD, Peterson ED, Dupre ME. Effectiveness of Telemedicine Visits in Reducing 30-Day Readmissions Among Patients With Heart Failure During the COVID-19 Pandemic. J Am Heart Assoc. 2022;11(7):e023935. doi: 10.1161/JAHA.121.023935 35229656 PMC9075458

[12] McAlister FA, Youngson E, Kaul P, Ezekowitz JA. Early Follow-Up After a Heart Failure Exacerbation: The Importance of Continuity. Circ Heart Fail. 2016;9(9):e003194. doi: 10.1161/CIRCHEARTFAILURE.116.003194 27618853

[13] Field TS, Ogarek J, Garber L, Reed G, Gurwitz JH. Association of early post-discharge follow-up by a primary care physician and 30-day rehospitalization among older adults. J Gen Intern Med. 2015;30(5):565–571. doi: 10.1007/s11606-014-3106-4 25451987 PMC4395599

[14] Dharmarajan K, Hsieh AF, Lin Z, Bueno H, Ross JS, Horwitz LI, et al. Diagnoses and timing of 30-day readmissions after hospitalization for heart failure, acute myocardial infarction, or pneumonia. Jama. 2013;309(4):355–363. doi: 10.1001/jama.2012.216476 23340637 PMC3688083

[15] Elixhauser A, Steiner C, Harris DR, Coffey RM. Comorbidity measures for use with administrative data. Med Care. 1998;36(1):8–27. doi: 10.1097/00005650-199801000-00004 9431328

[16] Baughman DJ, Jabbarpour Y, Westfall JM, Jetty A, Zain A, Baughman K, et al. Comparison of Quality Performance Measures for Patients Receiving In-Person vs Telemedicine Primary Care in a Large Integrated Health System. JAMA Netw Open.2022;5(9):e2233267. doi: 10.1001/jamanetworkopen.2022.33267 36156147 PMC9513647

[17] Zhang W, Cheng B, Zhu W, Huang X, Shen C. Effect of telemedicine on quality of care in patients with coexisting hypertension and diabetes: a systematic review and meta-analysis. Telemed J E Health. 2021;27(6):603–614. doi: 10.1089/tmj.2020.0122 32976084

[18] Timpel P, Oswald S, Schwarz PEH, Harst L. Mapping the evidence on the effectiveness of telemedicine interventions in diabetes, dyslipidemia, and hypertension: an umbrella review of systematic reviews and meta-analyses. J Med internet Res. 2020;22(3):e16791. doi: 10.2196/16791 32186516 PMC7113804

[19] Chongmelaxme B, Lee S, Dhippayom T, Saokaew S, Chaiyakunapruk N, Dilokthornsakul P. The effects of telemedicine on asthma control and patients’ quality of life in adults: a systematic review and meta-analysis. J Allergy Clin Immunol Pract. 2019;7(1):199–216.e11. doi: 10.1016/j.jaip.2018.07.015 30055283

[20] Wakefield BJ, Holman JE, Ray A, Scherubel M, Adams MR, Hillis SL, et al. Effectiveness of home telehealth in comorbid diabetes and hypertension: a randomized, controlled trial. Telemed J E Health. 2011;17(4):254–261. doi: 10.1089/tmj.2010.0176 21476945

[21] Wakefield BJ, Holman JE, Ray A, Scherubel M, Adams MR, Hills SL, Rosenthal GE. Outcomes of a home telehealth intervention for patients with diabetes and hypertension. Telemed J E Health. 2012;18(8):575–579. doi: 10.1089/tmj.2011.0237 22873700

[22] Baughman D, Ptasinski A, Baughman K, Buckwalter N, Jabbarpour Y, Waheed A. Comparable quality performance of acute low-back pain care in telemedicine and office-based cohorts. Telemed J E Health. Published online March 28, 2022. doi: 10.1089/tmj.2021.0535 35349350

[23] Shaw JA, Stiliannoudakis S, Qaiser R, Layman E, Sima A, Ali A. Thirty-Day Hospital Readmissions: A Predictor of Higher All-cause Mortality for Up to Two Years. Cureus. 2020 Jul 21;12(7):e9308. doi: 10.7759/cureus.9308 . 32839677 PMC7440272

